# Distribution of citalopram enantiomers following medication with racemic citalopram - a naturalistic therapeutic drug monitoring study

**DOI:** 10.1007/s00228-026-04148-x

**Published:** 2026-07-24

**Authors:** Ketil Arne Espnes, Arne Hønnås, Eirik Skogvoll, Olav Spigset

**Affiliations:** 1 Department of Clinical Pharmacology, St. Olavs Hospital, Trondheim University Hospital, Torgarden, P.O. Box 3250, 7006 Trondheim, Norway; 2 Clinic of Anaesthesia and Intensive Care, St. Olavs Hospital, Trondheim University Hospital, Trondheim, Norway; 3 Department of Circulation and Medical Imaging (ISB), Faculty of Medicine and Health Sciences, Norwegian University of Science and Technology (NTNU), Trondheim, Norway; 4 Department of Clinical and Molecular Medicine (IKOM), Faculty of Medicine and Health Sciences, Norwegian University of Science and Technology (NTNU), Trondheim, Norway

**Keywords:** Citalopram, Racemic intake, Enantiomers, Pharmacokinetics

## Abstract

**Purpose:**

There are very few reports describing to which extent sex, age, dose and time from dose intake affect the enantiomeric distribution during treatment with citalopram. The aim of the present study was to quantify the impact of these factors on the active S-enantiomer during treatment with citalopram.

**Methods:**

Samples analysed for citalopram in a routine therapeutic drug monitoring setting were included. The analytical method consisted of enantiomeric separation and quantification of R- and S-citalopram. We employed a linear mixed model to account for multiple samples from the same patient.

**Results:**

A total of 305 samples was included. The mean (± SD) proportion of S-citalopram in the total sample was 34.2 ± 8.1%, corresponding to a mean S/R ratio of 0.52 ± 0.17. According to the linear mixed model, the proportion of S-citalopram increased with 0.69 percentage points per decade of age. Increasing the time interval from dose intake to sampling reduced this proportion with 1.10 percentage points every 6 h. For example, if the sample was obtained 24 h after intake in a person aged 40, the proportion of S-citalopram was 32.7%; while if the sample was obtained 12 h after intake the proportion was 34.9%. Sex and dose did not impact significantly.

**Conclusion:**

The percentage of S-citalopram in plasma during treatment with citalopram increased with patient age and decreased with the time from dose intake. In contrast, sex and dose did not significantly affect the percentage of S-citalopram.

## Background

The selective serotonin reuptake inhibitor (SSRI) citalopram has been used in the treatment of depression and other mental disorders for several decades. Citalopram is a racemate, constituting of a 50:50 mixture of the R-citalopram and S-citalopram and with S-citalopram being the active enantiomer. In Norway, citalopram was marketed in 1995 whereas the pure S-enantiomer, escitalopram, was marketed in 2002. In 2005, escitalopram surpassed citalopram as the most prescribed SSRI. Citalopram continued to be the second most prescribed SSRI from 2005 to 2012 and was thereafter the third most prescribed after escitalopram and sertraline [1]. This trend, with a rise in escitalopram consumption and a decrease in citalopram consumption over the years, corresponds to what has been reported internationally [2, 3]. The reason for this shift is probably mainly related to marketing issues, as most current evidence suggests that there are no clinically relevant differences in therapeutic effect or in adverse drug reaction profile between the two drugs [4, 5]. Citalopram is advocated as the preferred drug in regions where the cost is lower than for escitalopram [5, 6] and its use is still significant world-wide [2, 3].

The major enzymes involved in the metabolism of the citalopram enantiomers are CYP2C19 and CYP3A4, with a smaller contribution from CYP2D6 [7–9]. Based on studies in human liver microsomes, the most important difference between the enantiomers is that CYP2C19 is of particular importance for the metabolism of S-citalopram, whereas its role is somewhat less, although still significant, for R-citalopram [8, 9]. Oral clearance is higher and the elimination half-life is shorter for S-citalopram than for R-citalopram, with a mean ± standard deviation (SD) half-life of 35 ± 4 h for S-citalopram and 47 ± 11 h for R-citalopram reported in one study [10]. These pharmacokinetic differences also explain the relatively low S/R ratios reported for citalopram, varying from 0.59 ± 0.13 to 0.7 ± 0.1 at steady state in samples obtained at trough 12–24 h after drug intake [10–13]. These values correspond to an S-citalopram proportion of 37–41% of total citalopram. The higher clearance of S-citalopram is most likely attributable to stereoselective differences in the affinity to the enzymes and the metabolic capacity of the enzymes involved [7–9].

There are several studies examining the effects of sex and age on the total concentrations of citalopram, showing increased dose-corrected concentrations in advanced age but an inconsistent effect of sex [14–17]. In contrast, data on the possible influence of sex and age on the distribution of the two enantiomers is sparse. In a population pharmacokinetic study in patients with Alzheimer’s disease aged 47–90 (mean 77.8) years the clearance of both S- and R-citalopram decreased with age, whereas the clearance of the R but not the S enantiomer was higher in men than in women [18]. In another study in eight elderly patients, S/R ratios were higher than those previously reported in younger subjects [13]. The role of citalopram dose on the S/R ratio is inconclusive. In an experimental study in rats the S/R ratio decreased with increased citalopram dosage [19], and a therapeutic drug monitoring (TDM) study reported that the S/R ratio decreased with increasing doses [20].

There are very few reports describing to which extent sex, age, dose and time interval between dose intake and sampling affect the enantiomeric distribution of citalopram. The aim of the present study was to quantify the impact of these factors on the S- and R-enantiomers during treatment with citalopram.

## Materials and methods

### Participants and sampling

Data for specimens analysed for citalopram in our laboratory between July 25th, 2017, and February 27th, 2020, was drawn from our routine TDM database. This period was chosen because it was the time span from the introduction of an enantiomer-specific routine method for the analysis of citalopram at our laboratory [21] until the introduction of a new TDM data management system. The study was approved by the Regional Committee for Health Research Ethics of Central Norway (approval No. 2021/271726).

**Fig. 1:**
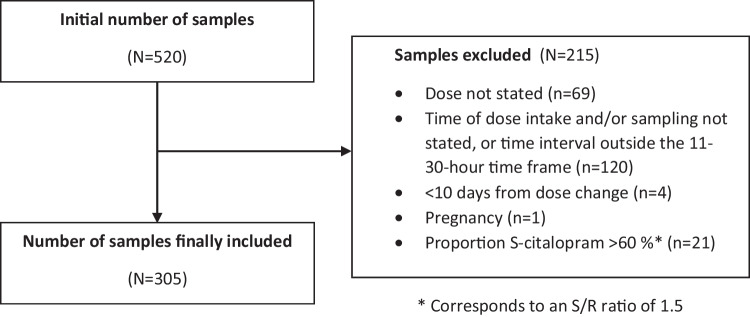
Flowsheet of the inclusion and exclusion of samples from the collected data

From the 520 samples retrieved from the database, 305 were included for statistical analyses (Fig. 1). The exclusion of samples with an S-citalopram percentage above 60 (which corresponds to an S/R ratio of 1.5) was based on two factors. According to our own TDM experience such high percentages are caused by either a combined intake of escitalopram and citalopram or that the patient has recently switched between the drugs. In addition, results from studies in CYP2C19 poor metabolizers, and in subjects being concomitantly treated with CYP2C19 inhibitors (both which would be expected to achieve the highest S/R ratios) show that none of these subjects reached an S-citalopram percentage above 60. In most cases they had percentages around 50, corresponding to an S/R ratio of close to 1 [22, 23].

### Drug analysis

The samples were analysed with a method for enantiomeric separation and quantification of R- and S-citalopram in serum using ultra-high performance supercritical fluid chromatography-tandem mass spectrometry (UHPSFC-MS/MS). The method was developed and validated in our laboratory and has been published in detail previously [21]. The laboratory is accredited by Norwegian Accreditation according to NS-EN ISO 15189:2022 within the areas M04 (Clinical Pharmacology), M30 (Specimen Collection) and M31 (Flexible scope of accreditation). In brief, sample preparation prior to analysis consisted of protein precipitation and filtration through a phospholipid removal plate. R/S-citalopram-d_4_ and S-citalopram-d_6_ were used as internal standards. Separation was performed with a UPC2 Trefoil CEL2 column (Waters, Taunton, MA, USA) and the mobile phase consisted of CO_2_ and methanol/acetonitrile with ammonium acetate. Detection was performed on a Xevo TQ-S tandem-quadropole mass spectrometer (Waters, Manchester, UK) with positive electrospray ionization and two multiple reaction monitoring transitions (m/z 325.1 > 262.0 and m/z 325.1 > 109.0). The calibration ranges were 5–500 nmol/L for each of the two enantiomers and the between-assay relative standard deviations were in the range of 3.4–4.5%. There is no indication that citalopram undergoes spontaneous or enzymatic chiral inversion [9]. In proficiency testing, z-scores obtained with the method have ranged from − 0.2 to + 0.7.

### Statistical procedures

The primary outcome variable was the concentration of S-citalopram relative to the concentration of total (R + S) citalopram, given in percent. In some previous studies, findings are reported as S/R ratios or R/S ratios. These ratios can be calculated from the percentage of S-citalopram by the following equations:

- S/R ratio = percentage S-citalopram/(100 – percentage S-citalopram)
- R/S ratio = (100 – percentage S-citalopram)/percentage S-citalopram

Basic data handling was performed in Microsoft Excel 2016. Descriptive data are given as means ± standard deviations (SD) or numbers and percentage, as appropriate. Further statistical analysis was done in SPSS version 29 and Stata version 19.5. We employed a linear mixed model for the outcome, with patient ID as a random factor, as many patients had more than one sample. More complex models were tested but did not provide meaningful improved fit to the data. The results are presented as scatter plots of individual results, along with expected values plus or minus one standard deviation. P-values < 0.05 were considered statistically significant.

## Results

A total of 305 samples from 237 patients were included in the statistical analyses (Fig. 1). Patient characteristics, citalopram doses and concentrations and other sampling variables are presented in Table 1.

**Table 1:** Number of patients included, patient characteristics, number of samples, citalopram doses, sampling times after last intake, concentrations and concentration/dose ratios (CDRs) of citalopram, S-citalopram and R- citalopram, and percent S-citalopram

	**Total**	**Males**	**Females**
Number of patients	237	61 (25.7%)	176 (74.3%)
Age at first sample (years), mean (SD), min–max	57.8 (19.5) 18—92	61.6 (17.8) 22—90	56.6 (19.8) 18—92
Number of samples	305	85 (27.9%)	220 (72.1%)
Citalopram dose (mg), mean (SD) min–max	25.0 (11.9) 5–60	25.5 (11.3) 10—60	24.8(12.1) 5—60
Time from last intake to sampling (hours), mean (SD), min–max^a^	21.7 (5.2) 11–30	21.9 (4.7) 11—29	21.6 (5.3) 11—30
Citalopram concentration (nmol/L), mean (SD), min–max	184 (121) 22–710	173 (113) 28–517	188 (124) 22—710
S-citalopram concentration (nmol/L), mean (SD), min–max	66.5 (53.3) 6–290	64.4 (49.9) 6—207	67.3 (54.7) 6—290
R-citalopram concentration (nmol/L), mean (SD), min–max	117 (72.4) 16–420	108 (67.0) 22—333	120 (74.2) 16—420
CDR citalopram ([nmol/L]/[mg/d]), mean (SD), min–max	7.79 (4.80) 1.38–30.0	7.22 (4.98) 1.40–25.9	8.01 (4.73) 1.38–30.0
CDR S-citalopram^b^ ([nmol/L]/[mg/d]), mean (SD), min–max	2.85 (2.22) 0.3–12.6	2.69 (2.22) 0.3–10.4	2.91 (2.23) 0.3–12.6
CDR R-citalopram^b^ ([nmol/L]/[mg/d]), mean (SD), min–max	4.94 (2.77) 0.88–18.0	4.53 (2.92) 1.10–16.7	5.10 (2.71) 0.88–18.0
Percent S-citalopram, mean (SD), min–max	34.2 (8.1) 14.9–52.9	35.3 (7.5) 20.5–51.8	33.8 (8.2) 14.9–52.9

CDR = concentration/dose ratio; SD = standard deviation.

^a^ According to information on the requisition forms, citalopram was administered once daily in all cases.

^b^ Calculated from the dose of racemic (S + R) citalopram.

**Fig. 2:**
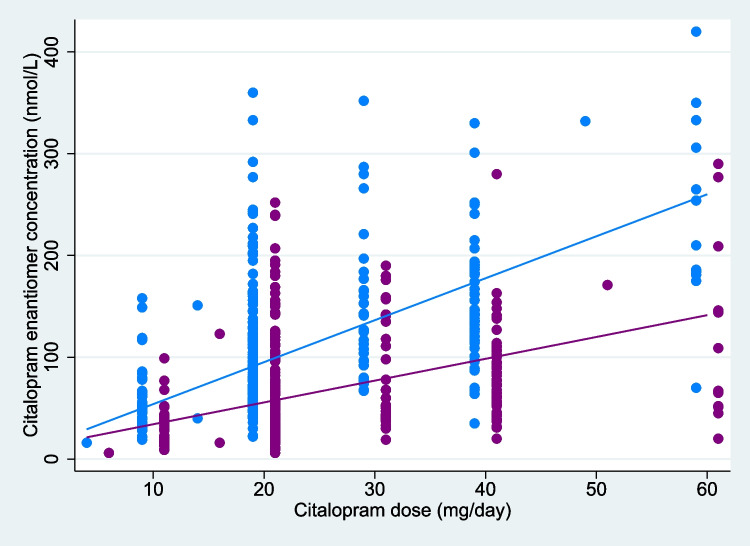
Concentrations of S-citalopram (dark [purple] symbols) and R-citalopram (light [blue] symbols) at different daily doses. To increase readability, the individual symbols for R-citalopram are shown slightly to the left of the doses used (and indicated on the x-axis), whereas the symbols for S-citalopram are shown to the right. As the concentrations of the R and the S enantiomers increase proportionally, the ratio between them, and thereby also the percentage of S-citalopram, remain unchanged irrespective of dose

The mean (± SD) proportion of S-citalopram in the total sample was 34.2 ± 8.1%, corresponding to a mean S/R ratio of 0.52 ± 0.17. The relationship between citalopram doses and concentrations of the two enantiomers is shown in Fig. 2.

Parameter estimates from the linear mixed model analysis are presented in Table 2. The intraclass correlation was estimated as 0.82, indicating a large inter-individual variability. There were no significant associations between dose or sex and percent S-citalopram. In contrast, there were a statistically significant positive relation between age and percent S-citalopram and a statistically significant negative relation between the time interval from dose intake and percent S-citalopram (Table 2, upper panel). After excluding the two non-significant variables (Table 2, lower panel), the principal results for age and for time from dose intake remained virtually unchanged.

**Table 2:** Factors potentially affecting the percentage of S-citalopram in 305 samples from 237 patients treated with citalopram, using a linear mixed effects model

Factor	Coefficient	95% confidence interval	P value
***Model 1 – all variables included***			
Intercept	35.19	30.29 to 40.08	< 0.001
Dose (per mg)	0.118	−0.052 to 0.076	0.72
Sex (female)	−1.47	−3.74 to 0.80	0.21
Age (per year)	0.065	0.013 to 0.117	0.015
Time from last dose to sampling (per hour)	−0.178	−0.325 to −0.031	0.018
***Model 2 – only statistically significant variables in Model 1 included***			
Intercept	34.29	30.22 to 38.35	< 0.001
Age (per year)	0.069	0.017–0.120	0.010
Time from last dose to sampling (per hour)	−0.183	−0.330 to −0.035	0.015

**Fig. 3:**
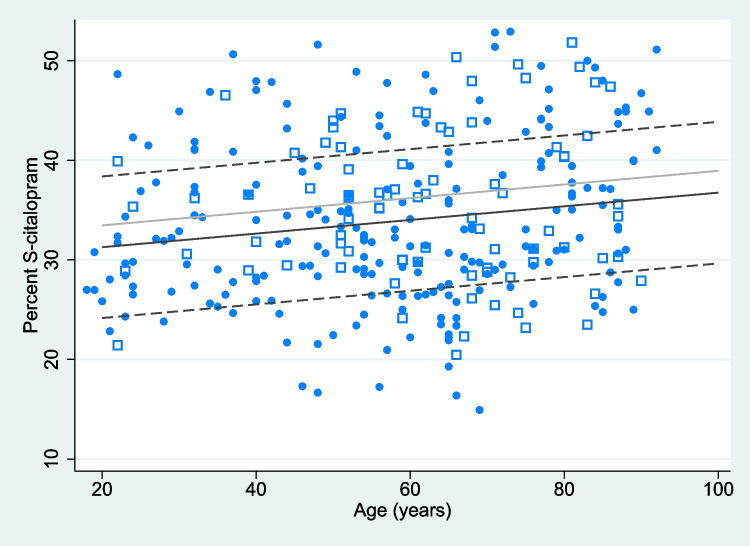
Scatterplot of observed percent S-citalopram as a function of age (males = open squares, women = filled circles) overlaid results from the linear mixed model. The black solid line represents the expected (“average”) value 24 h after last dose intake, while the dashed black lines represent patients that are 1 standard deviation above or below average. The grey line represents the expected value 12 h after last dose intake

Results from this final model are illustrated in Fig. 3.

According to the final model S-citalopram increased with 0.69 percentage points per decade, increasing the proportion S-citalopram from 31.3% at the age 20 years to 35.4% at the age 80 years in samples obtained 24 h after dose intake. Increasing the time interval from dose intake to sampling caused a decrease in S-citalopram by 1.10 percent points for every 6 h. As an example, shortening the sampling interval from 24 to 12 h in a person aged 40 will increase the proportion S-citalopram from 32.7% to 34.9%.

## Discussion

The principal finding in the present study is that the percentage of S-citalopram in plasma during treatment with citalopram increased with patient age and decreased with the time interval from dose intake to sampling. In contrast, citalopram dose and patient sex did not significantly affect the percentage of S-citalopram.

The crude mean proportion S-citalopram of 34.2% in our study is somewhat lower than the 37–41% interval reported in previous studies [10–13]. Our analysis is consistent with the crude mean, with proportions of 32.7% 24 h after dose intake and 34.9% 12 h after dose intake in a 40-year-old person. The age effect, where S-citalopram increased with 0.69 percentage points per decade of age is most likely due to an age-related differential effect of clearance of the two enantiomers. Both CYP2C19 and CYP3A4 activities have been found to diminish with increasing age [24–26] and it has been shown for citalopram that the clearance of both enantiomers decreased with age [18]. The only previously published study directly aiming to address the effect of age on enantiomer distribution found a mean S/R ratio of 0.65 (corresponds to about 40% S-citalopram) in eight patients with a mean age of 77 years [13]. However, they did not have their own control group and with only eight patients included, the mean value in their study could well be a result of random variations. According to our analysis, even though the mean proportion of S-citalopram in an 80-year-old person is 35.4% 24 h after dose intake, the + 1 SD value is above 40% (Fig. 3).

Increasing the time interval from dose intake to sampling caused a decrease in S-citalopram with 1.10 percentage points for every 6 h in our study. We are not aware of any previous study quantifying this time-dependency, but the effect may be readily explained pharmacokinetically, as the clearance is higher and the elimination half-life shorter for the S-enantiomer than for the R-enantiomer [10]. Our findings do not warrant any modification of the current TDM recommendation that samples should preferably be obtained at trough, but they show that when sampling is performed at non-standardised time points, not only the total drug concentration, but also the enantiomeric distribution, will be altered. In this perspective, it should be acknowledged that the therapeutic reference range for citalopram is not firmly established. The international guidelines by Hiemke et al. [27] recommend a reference range of 50–110 ng/mL for citalopram, whereas Norwegian national consensus has defined a reference interval of 70–350 nmol/L (23–114 ng/mL). For escitalopram, the corresponding ranges are 15–80 ng/mL and 20–120 nmol/L (7–39 ng/mL), respectively.

We did not find a statistically significant sex difference, although there was a trend towards a possibly higher percentage S-citalopram in males. It has been shown that CYP3A4 has a higher activity in females [28–31], whereas there has not been predicted any general sex differences in CYP2C19 or CYP2D6 activities [24, 30]. It has however been indicated that fertile females have a slightly higher CYP2D6 activity than males, and females using oral contraceptive may have a reduced CYP2C19 activity [32]. In a population pharmacokinetic study in 81 patients with Alzheimer’s disease aged 47–90 (mean 77.8) years, clearance of the R-enantiomer, but not the S-enantiomer, was higher in men than in women [18]. This effect is similar to the possible trend in our study, but the impact was small and any effects of sex on the enantiomeric distribution of citalopram are most likely negligible from a clinical point of view.

We did not find any effect of citalopram dose on the percentage S-citalopram, consistent with a situation where both enantiomers are undergoing first-order pharmacokinetics. The only study previously investigating this topic found an S/R ratio of 0.50 in those prescribed a daily dose of 20–30 mg citalopram, compared to 0.41 in those prescribed 80–100 mg/d [20]. When including any of the of the two intermediate dose range groups, 40–50 mg/d and 60–70 mg/d in the comparisons, statistical significance was no longer achieved. Our results did not reveal even a trend of any dose effect on the percentage S-citalopram. The reason might be that the doses used in our study was lower, 5–60 mg/d, with a mean dose of 25 mg/d. This dose range reflects the doses used in clinical practice today, after the recommended maximum dose of citalopram was lowered due to the effect of high doses on the QT interval in the electrocardiogram, with a subsequent risk of cardiac arrhythmias [33, 34].

Some limitations of our study warrant consideration. The naturalistic setting using TDM data means that we had to rely on the data given in the requisition forms accompanying the samples. We had no data on drug adherence, ethnic background, CYP genotypes, liver function, concomitant diseases or use of herbal medicines, and we had no means of verifying the information on the requisition forms regarding dosing and timing of last drug intake vs. sampling. As information related to comedication is often incomplete and unreliable, we decided not to study the influence of concomitant intake of CYP2C19/CYP3A4 inducers or inhibitors. However, based on previous data [23], it could be expected that those treated with CYP2C19 inhibitors would have an increased percentage of S-citalopram also in our material. Smoking data could also potentially have been of interest [35] but was not available. Finally, although a total of 305 samples is a relatively high number compared to previous studies, results could have been even more conclusive with more samples included.

In conclusion, when administering citalopram as a racemate, only about 30–40% comprise S-citalopram at steady state conditions. The percentage increased with age and decreased with time interval from last dose intake. In contrast, we did not reveal any influence of sex or dose. Finally, it should be emphasized that although the percentage of S-citalopram (or the S/R enantiomeric ratio) clearly has interest from a pharmacokinetic point of view, the primary determinant of clinical efficacy is the absolute plasma concentration of the pharmacologically active S-enantiomer and not the percentage or the ratio as such.

## Data Availability

No datasets were generated or analysed during the current study.

## References

[1] Norwegian Prescribed Drug Registry (NorPD). In: ed. Norwegian Institute of Public Health.

[2] Cavanah LR, Ray PK, Goldhirsh JL, Huey LY, Piper BJ (2025) Patterns in (es)citalopram prescriptions to Medicaid and Medicare patients in the United States: the potential effects of evergreening. Front Psychiatry 16:1450111. 10.3389/fpsyt.2025.1450111 40109440 10.3389/fpsyt.2025.1450111 PMC11919847

[3] Samardžić J, Simović F, Sekanić K, Branković M (2025) Five-year trends in SSRI consumption: a precision medicine approach to comparative analysis between Serbia and European countries. Healthcare (Basel). 10.3390/healthcare13101174 40428010 10.3390/healthcare13101174 PMC12111178

[4] Trkulja V (2010) Is escitalopram really relevantly superior to citalopram in treatment of major depressive disorder? A meta-analysis of head-to-head randomized trials. Croat Med J 51(1):61–73. 10.3325/cmj.2010.51.61 20162747 10.3325/cmj.2010.51.61 PMC2829184

[5] Escitalopram vs. citalopram: one enantiomer’s dominance reflects marketing not evidence (Therapeutics Letter 159). In: ed. Therapeutics Initiative. The University of British Columbia.

[6] Ong D, Ladhar S, Perry T, Carney G, Thompson W, Salzwedel D, Tejani AM (2026) Rising escitalopram use in British Columbia: is it rational and evidence-informed? Can J Psychiatry 71(5):406–408. 10.1177/07067437251405975 41452043 10.1177/07067437251405975 PMC13092817

[7] Rochat B, Amey M, Gillet M, Meyer UA, Baumann P (1997) Identification of three cytochrome P450 isozymes involved in N-demethylation of citalopram enantiomers in human liver microsomes. Pharmacogenetics 7(1):1–10. 10.1097/00008571-199702000-00001 9110356 10.1097/00008571-199702000-00001

[8] Olesen OV, Linnet K (1999) Studies on the stereoselective metabolism of citalopram by human liver microsomes and cDNA-expressed cytochrome P450 enzymes. Pharmacology 59(6):298–309. 10.1159/000028333 10575324 10.1159/000028333

[9] von Moltke LL, Greenblatt DJ, Giancarlo GM, Granda BW, Harmatz JS, Shader RI (2001) Escitalopram (S-citalopram) and its metabolites in vitro: cytochromes mediating biotransformation, inhibitory effects, and comparison to R-citalopram. Drug Metab Dispos 29(8):1102–1109 11454728

[10] Sidhu J, Priskorn M, Poulsen M, Segonzac A, Grollier G, Larsen F (1997) Steady-state pharmacokinetics of the enantiomers of citalopram and its metabolites in humans. Chirality 9(7):686–692. 10.1002/(sici)1520-636x(1997)9:7<686::Aid-chir9>3.0.Co;2-5 9366029 10.1002/(SICI)1520-636X(1997)9:7<686::AID-CHIR9>3.0.CO;2-5

[11] Rochat B, Amey M, Baumann P (1995) Analysis of enantiomers of citalopram and its demethylated metabolites in plasma of depressive patients using chiral reverse-phase liquid chromatography. Ther Drug Monit 17(3):273–279. 10.1097/00007691-199506000-00011 7624924 10.1097/00007691-199506000-00011

[12] Rochat B, Amey M, Van Gelderen H, Testa B, Baumann P (1995) Determination of the enantiomers of citalopram, its demethylated and propionic acid metabolites in human plasma by chiral HPLC. Chirality 7(6):389–395. 10.1002/chir.530070602 7577348 10.1002/chir.530070602

[13] Foglia JP, Pollock BG, Kirshner MA, Rosen J, Sweet R, Mulsant B (1997) Plasma levels of citalopram enantiomers and metabolites in elderly patients. Psychopharmacol Bull 33(1):109–112 9133760

[14] de Mendonça Lima CA, Baumann P, Brawand-Amey M, Brogli C, Jacquet S, Cochard N, Powell-Golay K, Eap CB (2005) Effect of age and gender on citalopram and desmethylcitalopram steady-state plasma concentrations in adults and elderly depressed patients. Prog Neuropsychopharmacol Biol Psychiatry 29(6):952–956. 10.1016/j.pnpbp.2005.06.001 16006029 10.1016/j.pnpbp.2005.06.001

[15] Fudio S, Borobia AM, Piñana E, Ramírez E, Tabarés B, Guerra P, Carcas A, Frías J (2010) Evaluation of the influence of sex and CYP2C19 and CYP2D6 polymorphisms in the disposition of citalopram. Eur J Pharmacol 626(2–3):200–204. 10.1016/j.ejphar.2009.10.007 19840783 10.1016/j.ejphar.2009.10.007

[16] Unterecker S, Riederer P, Proft F, Maloney J, Deckert J, Pfuhlmann B (2013) Effects of gender and age on serum concentrations of antidepressants under naturalistic conditions. J Neural Transm (Vienna) 120(8):1237–1246. 10.1007/s00702-012-0952-2 23254926 10.1007/s00702-012-0952-2

[17] Tveit K, Hermann M, Nilsen RM, Wallerstedt SM, Rongve A, Molden E, Hole K (2024) Age of onset for increased dose-adjusted serum concentrations of antidepressants and association with sex and genotype: an observational study of 34,777 individuals. Eur J Clin Pharmacol 80(3):435–444. 10.1007/s00228-023-03611-3 38197945 10.1007/s00228-023-03611-3 PMC10873233

[18] Akil A, Bies RR, Pollock BG, Avramopoulos D, Devanand DP, Mintzer JE, Porsteinsson AP, Schneider LS, Weintraub D, Yesavage J, Shade DM, Lyketsos CG (2016) A population pharmacokinetic model for R- and S-citalopram and desmethylcitalopram in Alzheimer’s disease patients with agitation. J Pharmacokinet Pharmacodyn 43(1):99–109. 10.1007/s10928-015-9457-6 26611790 10.1007/s10928-015-9457-6 PMC4720707

[19] Kugelberg FC, Apelqvist G, Carlsson B, Ahlner J, Bengtsson F (2001) In vivo steady-state pharmacokinetic outcome following clinical and toxic doses of racemic citalopram to rats. Br J Pharmacol 132(8):1683–1690. 10.1038/sj.bjp.0704015 11309239 10.1038/sj.bjp.0704015 PMC1572733

[20] Tanum L, Strand LP, Refsum H (2010) Serum concentrations of citalopram–dose-dependent variation in R- and S-enantiomer ratios. Pharmacopsychiatr 43(5):190–193. 10.1055/s-0030-1254106 10.1055/s-0030-1254106 20503148

[21] Hegstad S, Havnen H, Helland A, Falch BMH, Spigset O (2017) Enantiomeric separation and quantification of citalopram in serum by ultra-high performance supercritical fluid chromatography-tandem mass spectrometry. J Chromatogr B Analyt Technol Biomed Life Sci 1061–1062:103–109. 10.1016/j.jchromb.2017.07.009 28715684 10.1016/j.jchromb.2017.07.009

[22] Herrlin K, Yasui-Furukori N, Tybring G, Widén J, Gustafsson LL, Bertilsson L (2003) Metabolism of citalopram enantiomers in CYP2C19/CYP2D6 phenotyped panels of healthy Swedes. Br J Clin Pharmacol 56(4):415–421. 10.1046/j.1365-2125.2003.01874.x 12968986 10.1046/j.1365-2125.2003.01874.x PMC1884377

[23] Rocha A, Coelho EB, Sampaio SA, Lanchote VL (2010) Omeprazole preferentially inhibits the metabolism of (+)-(S)-citalopram in healthy volunteers. Br J Clin Pharmacol 70(1):43–51. 10.1111/j.1365-2125.2010.03649.x 20642546 10.1111/j.1365-2125.2010.03649.x PMC2909806

[24] Bebia Z, Buch SC, Wilson JW, Frye RF, Romkes M, Cecchetti A, Chaves-Gnecco D, Branch RA (2004) Bioequivalence revisited: influence of age and sex on CYP enzymes. Clin Pharmacol Ther 76(6):618–627. 10.1016/j.clpt.2004.08.021 15592333 10.1016/j.clpt.2004.08.021

[25] Scripture CD, Sparreboom A, Figg WD (2005) Modulation of cytochrome P450 activity: implications for cancer therapy. Lancet Oncol 6(10):780–789. 10.1016/s1470-2045(05)70388-0 16198984 10.1016/S1470-2045(05)70388-0

[26] Rattanacheeworn P, Kerr SJ, Kittanamongkolchai W, Townamchai N, Udomkarnjananun S, Praditpornsilpa K, Thanusuwannasak T, Udomnilobol U, Jianmongkol S, Ongpipattanakul B, Prueksaritanont T, Avihingsanon Y, Chariyavilaskul P (2021) Quantification of CYP3A and drug transporters activity in healthy young, healthy elderly and chronic kidney disease elderly patients by a microdose cocktail approach. Front Pharmacol 12:726669. 10.3389/fphar.2021.726669 34603040 10.3389/fphar.2021.726669 PMC8486002

[27] Hiemke C, Bergemann N, Clement HW, Conca A, Deckert J, Domschke K, Eckermann G, Egberts K, Gerlach M, Greiner C, Gründer G, Haen E, Havemann-Reinecke U, Hefner G, Helmer R, Janssen G, Jaquenoud E, Laux G, Messer T, Mössner R, Müller MJ, Paulzen M, Pfuhlmann B, Riederer P, Saria A, Schoppek B, Schoretsanitis G, Schwarz M, Gracia MS, Stegmann B, Steimer W, Stingl JC, Uhr M, Ulrich S, Unterecker S, Waschgler R, Zernig G, Zurek G, Baumann P (2018) Consensus guidelines for therapeutic drug monitoring in neuropsychopharmacology: update 2017. Pharmacopsychiatry 51(1–02):9–62. 10.1055/s-0043-116492 28910830 10.1055/s-0043-116492

[28] Hunt CM, Westerkam WR, Stave GM (1992) Effect of age and gender on the activity of human hepatic CYP3A. Biochem Pharmacol 44(2):275–283. 10.1016/0006-2952(92)90010-g 1642641 10.1016/0006-2952(92)90010-g

[29] Wolbold R, Klein K, Burk O, Nüssler AK, Neuhaus P, Eichelbaum M, Schwab M, Zanger UM (2003) Sex is a major determinant of CYP3A4 expression in human liver. Hepatology 38(4):978–988. 10.1053/jhep.2003.50393 14512885 10.1053/jhep.2003.50393

[30] Scandlyn MJ, Stuart EC, Rosengren RJ (2008) Sex-specific differences in CYP450 isoforms in humans. Expert Opin Drug Metab Toxicol 4(4):413–424. 10.1517/17425255.4.4.413 18524030 10.1517/17425255.4.4.413

[31] Thangavel C, Boopathi E, Shapiro BH (2013) Inherent sex-dependent regulation of human hepatic CYP3A5. Br J Pharmacol 168(4):988–1000. 10.1111/j.1476-5381.2012.02222.x 22994453 10.1111/j.1476-5381.2012.02222.x PMC3631386

[32] Hägg S, Spigset O, Dahlqvist R (2001) Influence of gender and oral contraceptives on CYP2D6 and CYP2C19 activity in healthy volunteers. Br J Clin Pharmacol 51(2):169–173. 10.1111/j.1365-2125.2001.01328.x 11259990 10.1111/j.1365-2125.2001.01328.x PMC2014435

[33] Citalopram and escitalopram: QT interval prolongation. In: ed. Drug Safety Update. United Kingdom Medicines and Healthcare Products Regulatory Agency (GOV.UK).

[34] Citalopram and heart problems - changes to recommended doses. In: ed. Safety Alerts; Safety Advisory. Therapeutic Goods Administration, Department of Health, Disability and Ageing, Australian Government.

[35] Scherf-Clavel M, Samanski L, Hommers LG, Deckert J, Menke A, Unterecker S (2019) Analysis of smoking behavior on the pharmacokinetics of antidepressants and antipsychotics: evidence for the role of alternative pathways apart from CYP1A2. Int Clin Psychopharmacol 34(2):93–100. 10.1097/yic.0000000000000250 30557209 10.1097/YIC.0000000000000250

